# The protective effect of the anti-Toll-like receptor 9 antibody against acute cytokine storm caused by immunostimulatory DNA

**DOI:** 10.1038/srep44042

**Published:** 2017-03-07

**Authors:** Yusuke Murakami, Ryutaro Fukui, Yuji Motoi, Takuma Shibata, Shin-Ichiroh Saitoh, Ryota Sato, Kensuke Miyake

**Affiliations:** 1 Division of Innate Immunity, Department of Microbiology and Immunology, The Institute of Medical Science, The University of Tokyo, 4-6-1 Shirokanedai, Minato-ku, Tokyo 108-8639, Japan; 2 Laboratory of Innate Immunity, Center for Experimental Medicine and Systems Biology, The Institute of Medical Science, The University of Tokyo, 4-6-1 Shirokanedai, Minato-ku, Tokyo 108-8639, Japan

## Abstract

Toll-like Receptor 9 (TLR9) is an innate immune receptor recognizing microbial DNA. TLR9 is also activated by self-derived DNA, such as mitochondrial DNA, in a variety of inflammatory diseases. We show here that TLR9 activation *in vivo* is controlled by an anti-TLR9 monoclonal Ab (mAb). A newly established mAb, named NaR9, clearly detects endogenous TLR9 expressed in primary immune cells. The mAb inhibited TLR9-dependent cytokine production *in vitro* by bone marrow-derived macrophages and conventional dendritic cells. Furthermore, NaR9 treatment rescued mice from fulminant hepatitis caused by administering the TLR9 ligand CpGB and D-(+)-galactosamine. The production of proinflammatory cytokines induced by CpGB and D-(+)-galactosamine was significantly impaired by the mAb. These results suggest that a mAb is a promising tool for therapeutic intervention in TLR9-dependent inflammatory diseases.

Toll-like Receptor 9 (TLR9) is an innate immune sensor that recognizes microbial DNA^1^. Ligand recognition of TLR9 is strictly controlled to avoid an unnecessary response to self-derived DNA such as mitochondrial DNA. If the controlling systems are disrupted, TLR9 senses self-derived DNA and induces inflammatory diseases. In autoimmune diseases such as systemic lupus erythematosus (SLE) and psoriasis, self-derived DNA forms complexes with various autoantibodies or anti-bacterial peptides^2^,^3^. In steady state, self-derived DNA is rapidly digested by DNases, but DNA in immune complexes is resistant to DNase and is delivered to endosomes/lysosomes via the B cell receptor^4^,^5^,^6^. TLR9 is localized in endosomes/lysosomes and responds to DNA in the immune complex, inducing the production of autoantibodies against DNA^7^. These studies demonstrate that TLR9 is a promising target for therapeutic intervention in autoimmune diseases. It has been reported that oligodeoxynucleotides that antagonize nucleic acid (NA)-sensing TLRs ameliorate autoimmune disease models of psoriasis and SLE^8^,^9^.

Previous studies have shown that TLR9 senses microbial DNA in endosomes/lysosomes and ligand-activated TLR9 traffic in the endolysosomal compartments^10^,^11^,^12^. Ligand-dependent TLR9 trafficking has a critical role in type I interferon (IFN) induction in macrophages and plasmacytoid dendritic cells (pDCs)^10^,^11^,^13^. These studies investigated the distribution of ligands, downstream signalling molecules or transfected TLR9. Endogenous TLR9 has not been studied because of the lack of a monoclonal antibody (mAb) capable of detecting endogenous TLR9 when performing confocal microscopy analysis.

Recently, we have established mAbs to mouse TLR3, TLR7 and TLR9^14^,^15^,^16^. These mAbs show that endogenous TLR3, TLR7 and TLR9 are detectable on the surface of macrophages and DCs and in endolysosomes. Moreover, the mAb to TLR3 augments TLR3 responses, whereas that to TLR7 inhibits TLR7 responses. These results indicate that mAbs to NA-sensing TLRs are promising tools to control immune responses driven by NA-sensing TLRs.

Unc93 homolog B1 (Unc93B1) is a multiple transmembrane protein controlling TLR7 responses^17^,^18^,^19^,^20^. In *Unc93b1*^D34A/D34A^ mice, TLR7 is hyperactivated, causing thrombocytopenia, splenomegaly and hepatitis^21^. Since the administration of the anti-TLR7 mAb can ameliorate TLR7-dependent systemic inflammation in *Unc93b1*^D34A/D34A^ mice^22^, the antibody bound to NA-sensing TLRs is expected to act as a tool for therapeutic approaches. Although TLR9 is another promising target of therapeutic intervention in autoimmune diseases, previously established anti-TLR9 mAbs fail to inhibit TLR9 responses. It is, therefore, currently unknown whether anti-TLR9 mAb can control TLR9-dependent inflammatory diseases.

In this report, we established a novel anti-mouse TLR9 mAb that functions in confocal microscopy and flow cytometry, and inhibits TLR9 responses *in vitro* and *in vivo*. Administration of the anti-TLR9 mAb protected mice from acute hepatitis caused by the TLR9 ligand and D-(+)-galactosamine, and these results demonstrate that the anti-TLR9 mAb is a promising tool to control TLR9-dependent inflammatory diseases.

## Results

### New anti-mouse TLR9 mAb recognizing the N-terminal fragment of TLR9

**Figure 1:**
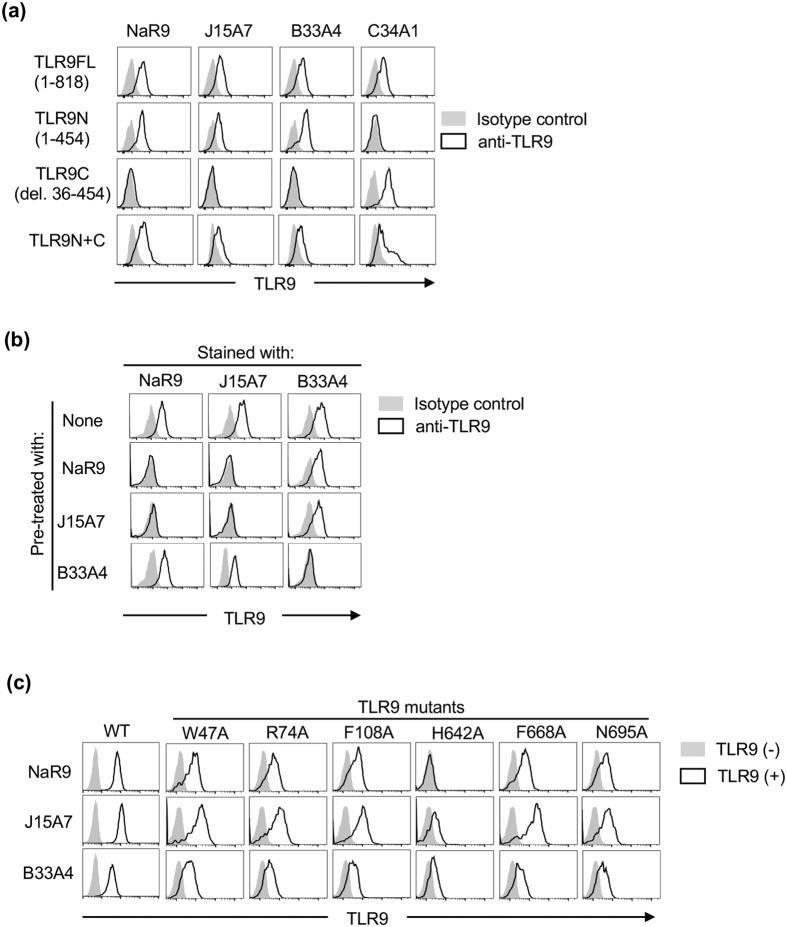
Establishment of anti-TLR9 mAb. (**a**) Ba/F3 cells expressing full-length TLR9 (FL:1–818), truncated TLR9N (1–454) or truncated TLR9C (deletion 36–454), or co-expressing TLR9N and TLR9C are subjected to membrane-permeabilized staining with NaR9, J15A7, B33A4 or C34A1 as indicated. (**b**) Ba/F3 cells expressing TLR9FL were subjected to membrane-permeabilized staining with biotinylated NaR9, C34A1 or PE-conjugated J15A7. Before staining with biotinylated mAbs, permeabilized cells were treated with unlabelled mAbs as indicated. (**c**) Ba/F3 cells expressing wild-type TLR9 (WT) or TLR9 mutants, including W47A, R74A, F108A, H642A, F668A and N695A, were subjected to membrane-permeabilized staining with NaR9, J15A7 and B33A4. (**a**,**b**) Grey histograms show staining with the isotype control (IgG1 or IgG2a). Open histograms indicate staining with an anti-TLR9 mAb. (**c**) All cells were stained with the indicated anti-TLR9 mAb. Grey histograms show the staining of cells without the TLR9-expressing plasmid. Open histograms show the staining of cells with the TLR9-expressing plasmid. These experiments were repeated at least twice, and representative data are shown.

We have generated three anti-mouse TLR9 mAbs, B33A4, C33A1 and J15A7^14^, but they do not clearly inhibit the TLR9 response. We tried to establish a new anti-TLR9 with an inhibitory effect on the TLR9 response and generated a new anti-TLR9 mAb named NaR9, classed as IgG2a/κ. To locate the epitope of NaR9, Ba/F3 cells expressing full length, N-terminal or C-terminal fragments of TLR9 were stained by NaR9 and the other anti-TLR9 mAbs. NaR9 specifically reacted with the N-terminal fragment of TLR9 (Fig. 1a), and a crossblocking study showed NaR9 bound to the region close to the epitope of J15A7 but not to the epitope of B33A4 (Fig. 1b).

For further analysis, we stained various TLR9s with mutations at the CpGB binding sites^23^. Among the mutants, the H642A mutation abolished NaR9 binding to TLR9 (Fig. 1c). Because the other two mAbs, J15A7 and B33A4, also impaired binding to the H642A mutant, TLR9 conformation may be altered by the H642A mutation. The other mutants did not alter NaR9 binding to TLR9, so the epitope of NaR9 might not be close to the CpGB binding sites.

### Endogenous TLR9 is detected by NaR9

**Figure 2:**
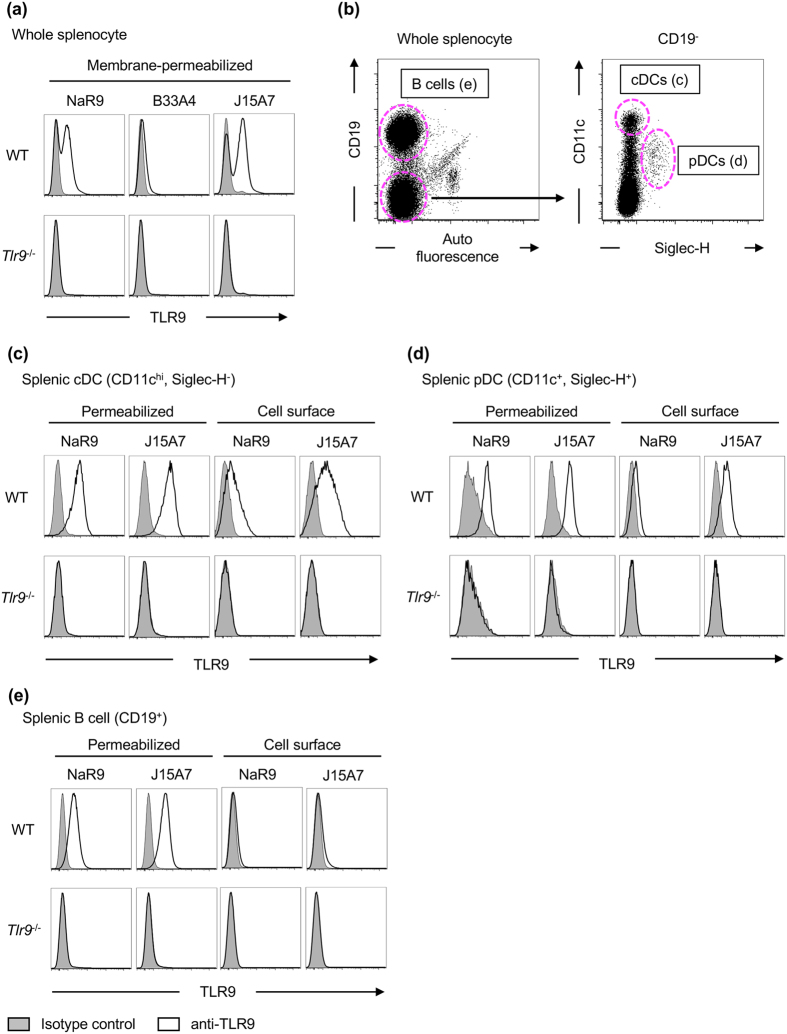
Cell surface and membrane-permeabilized staining of TLR9. (**a**–**e**) Spleen cells from WT or *Tlr9*^−/−^ mice were stained by anti-CD19, anti-CD11c and anti-SiglecH mAbs. Stained cells were subjected to staining with isotype controls (grey histograms) or anti-TLR9 mAbs (open histograms). TLR9 was stained with or without a membrane-permeabilizing reagent. (**a**) TLR9 expression in whole splenocytes with a membrane-permeabilizing reagent. (**b**) Gating strategy for analysis of B cells (left dot plot), cDCs and pDCs (right dot plot). TLR9 expression in cells surrounded by the dotted magenta line is shown in (**c–e**). At least 4 mice were used for each experiment, and independent experiments were repeated at least 3 times. Representative data are shown.

The expression of endogenous TLR9 in primary immune cells has not been extensively studied. To detect endogenous TLR9 in primary immune cells, we stained spleen cells from WT or *Tlr9*^−/−^ mice with 3 anti-TLR9 mAbs (Fig. 2a). NaR9 detected endogenous TLR9 in WT spleen cells but not in *Tlr9*^−/−^ spleen cells. B33A4 showed a much lower intensity of staining than NaR9. J15A7 showed the highest intensity of staining among the three mAbs tested. According to these results, NaR9 and J15A7 were used for further experiments.

**Figure 3:**
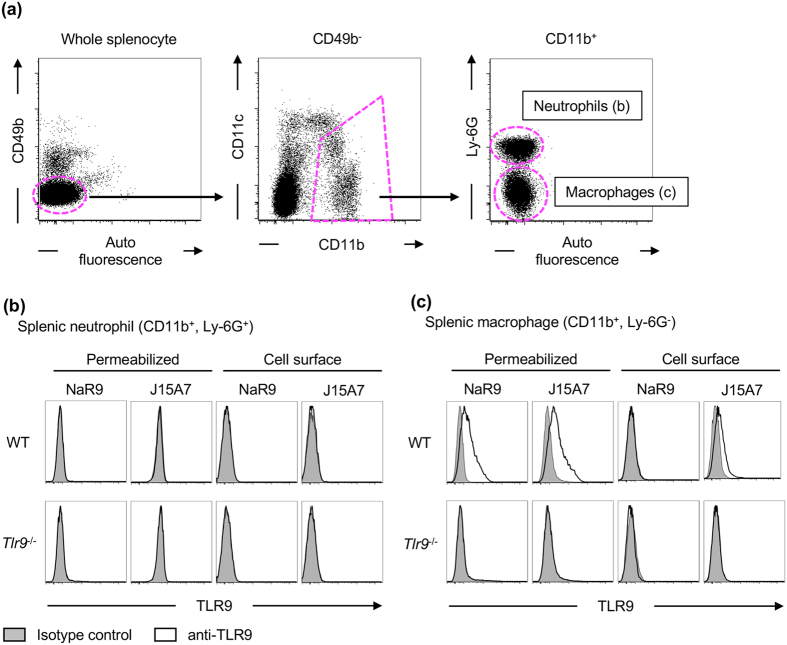
TLR9 is expressed in Ly-6G^−^ monocytes. (**a**–**c**) Whole splenocytes from WT or *Tlr9*^−/−^ mice were stained by anti-CD49b, anti-CD11b, anti-CD11c and anti-Ly-6G mAbs. Stained cells were subjected to staining with isotype controls (grey histograms) or anti-TLR9 mAbs (open histograms). TLR9 was stained with or without a membrane-permeabilizing reagent. (**a**) Gating strategy for the analysis of CD11b^+^ monocytes. To exclude CD11b^+^ NK cells, CD49b^+^ cells were gated out (left dot plot). TLR9 expression in cells surrounded by the dotted magenta line is shown in (**b**,**c**). At least 4 mice were used in each experiment, and independent experiments were repeated at least 3 times. Representative data are shown.

In a previous report, we showed that TLR9 is expressed on the surface of splenic conventional dendritic cells (cDCs) and plasmacytoid DCs (pDCs) as well as in intracellular vesicles of bone marrow-derived cDCs (BM-cDCs) and BM-pDCs by using J15A7^14^. In this report, we have improved the method of staining and analysed the TLR9 expression patterns again in various immune cells using NaR9 and J15A7. Both NaR9 and J15A7 detected TLR9 in splenic cDCs (CD11c^hi^, Siglec-H^−^) and pDCs (CD11c^+^, Siglec-H^+^) under the membrane permeabilizing condition (Fig. 2b–d). Cell surface TLR9 was also detected by these anti-TLR9 mAbs, although the staining intensity with NaR9 was lower than that with J15A7 (Fig. 2c,d). TLR9 is also detectable in B cells by membrane-permeabilized staining. However, cell surface TLR9 was hardly detected (Fib. 2b, e). Next, we studied TLR9 expression in splenic monocytes. CD11b^+^ Ly-6G^+^ neutrophils did not express TLR9, whereas CD11b^+^ CD49b^−^ Ly6G^−^ monocytes weakly but significantly expressed TLR9 (Fig. 3a–c). TLR9 was weakly detected on the surface of Ly-6G^−^ monocytes by J15A7 but was undetectable by NaR9 (Fig. 3c).

### The expression of TLR9 is increased in cells by Type I IFN stimulation

**Figure 4:**
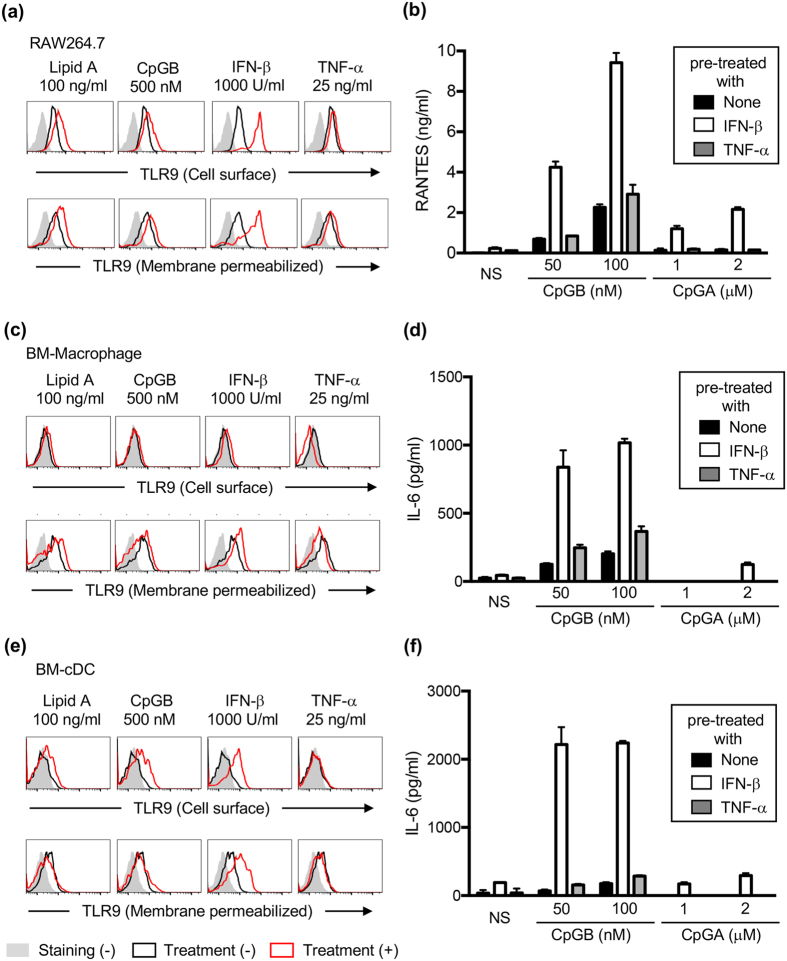
Expression of TLR9 is increased by IFN-β stimulation. (**a**,**c**,**e**) RAW264.7 cells (**a**), BM-Macs(**c**) and BM-cDCs (**e**) were treated with medium, lipid A (100 ng/ml), CpGB (500 nM), IFN-β (1000 U/ml) or TNF-α (25 ng/ml). Twenty-four hours later, cells were subjected to cell surface (upper panel) or membrane-permeabilized staining (lower panel). Grey histograms show staining with isotype control. Black and red open histograms show staining with NaR9 before and after treatment. (**b**,**d**,**f**) RAW264.7 cells (**b**), BM-Macs (**d**) and BM-cDCs (**f**) were pre-treated with medium, IFN-β (1000 U/ml) or TNF-α (25 ng/ml). Twenty-four hours later, the cells were stimulated with the indicated concentration of CpGB or CpGA. Twenty-four hours after stimulation with CpGB or CpGA, culture supernatants were collected, and the concentration of RANTES (**b**) or IL-6 (**d**,**f**) was determined by ELISA. The results are represented by the mean value and the s.d. from triplicate wells. These experiments were repeated at least twice, and representative data are shown.

The molecular mechanism controlling TLR9 expression was studied next. A macrophage cell line RAW264.7, bone marrow-derived macrophages (BM-Macs) and bone marrow-derived conventional DCs (BM-cDCs) were treated with lipid A, CpGB, IFN-β or TNF-α for 24 h, and cells were stained by NaR9 with or without membrane permeabilization. IFN-β strongly induced TLR9 expression, whereas lipid A, CpGB and TNF-α weakly increased TLR9 expression, (Fig. 4a,c,e). Interestingly, induction of TLR9 expression by IFN-β in BM-Macs was weaker than in RAW264.7 cells or BM-cDCs.

To investigate the correlation between TLR9 expression and TLR9 response, these cells were pre-treated with IFN-β or TNF-α for 24 h and then stimulated with the TLR9 ligands CpGB or CpGA. Pre-treatment with IFN-β enhanced the production of RANTES by TLR9 ligands in RAW264.7 cells (Fig. 4b) and enhanced the production of IL-6 in BM-Macs and BM-cDCs (Fig. 4d,f).

### Endogenous TLR9 localizes in late endosome and lysosome

**Figure 5:**
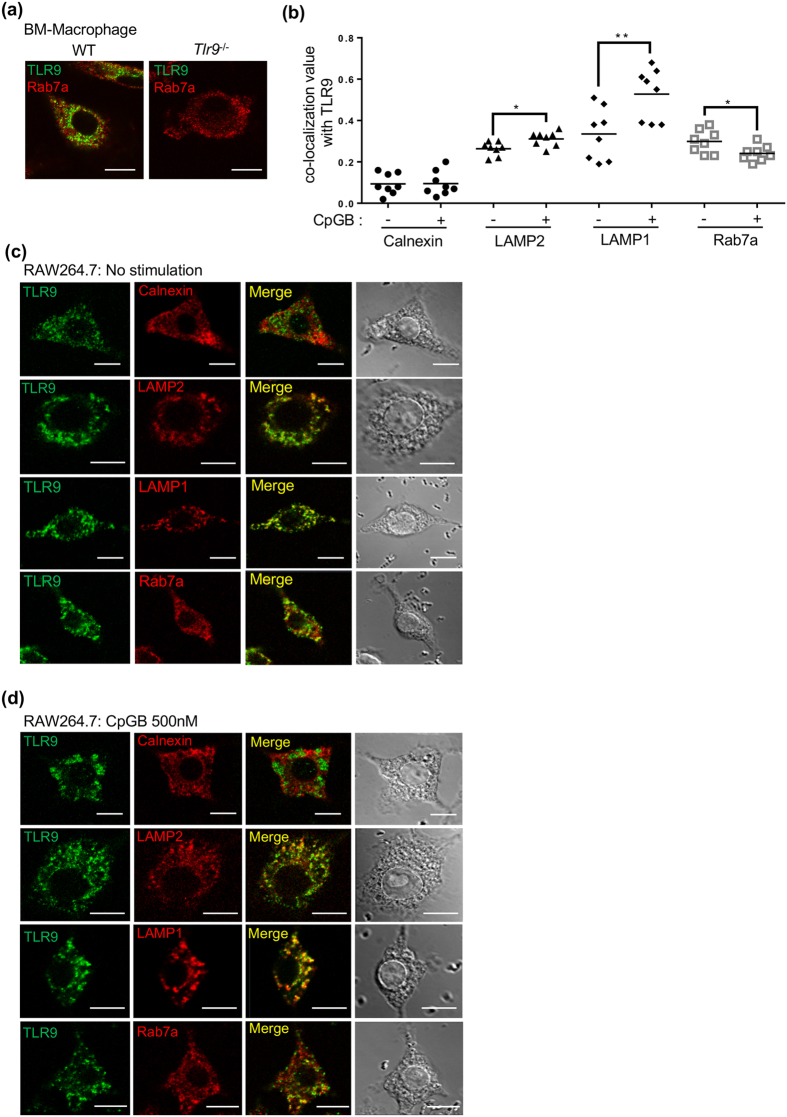
Endogenous TLR9 is localized in late-endosomes/lysosomes. (**a**) BM-Macs from WT or *Tlr9*^−/−^ mice were stained with NaR9. Rab7a was stained as a marker of late-endosomes. (**b–d**) RAW264.7 cells were stained with NaR9 and Abs to Calnexin, LAMP2, LAMP1 or Rab7a as a marker of ER, lysosomes or late-endosomes. (**b**) Pearson’s correlation coefficients were calculated using LSM710 ZEN software for the statistical analysis of co-localization and shown as absolute values. A Pearson’s coefficient of <0.2 represents poor co-localization. Each set of eight cells were used for statistical analysis. (**c**,**d**) Cells were incubated with or without CpGB (500 nM) for 24 h. Data were compared by performing a Student’s t-test: *p < 0.05, **p < 0.01 (**b**). These experiments were repeated at least twice, and representative data are shown. Scale bar, 10 μm.

TLR9 is synthesized in the endoplasmic reticulum (ER) and transported to the endosome or lysosome by a chaperone molecule Unc93B1^14^. Previous studies have shown the distribution of transfected and epitope-tagged TLR9^10^,^12^, but the distribution of endogenous TLR9 has not been reported. NaR9 was employed to detect endogenous TLR9 in BM-Macs, and endogenous TLR9 was specifically detected in WT but not detected in *Tlr9*^−/−^ BM-Macs. These data verify the specificity of NaR9 in confocal microscopy analysis (Fig. 5a).

Next, ligand-dependent trafficking of endogenous TLR9 was observed in RAW264.7 cells. Without CpGB stimulation, TLR9 colocalized with LAMP1, LAMP2 and Rab7a, markers for endosomes and lysosomes, but showed much less colocalization with Calnexin, an ER marker (Fig. 5b,c). After CpGB stimulation for 24 h, TLR9 colocalization with LAMP1 was significantly increased (Fig. 5b,d). TLR9 colocalization with LAMP2 was weakly increased, whereas TLR9 colocalization with a late endosome marker Rab7a was weakly decreased. These results suggest that ligand stimulation transports endogenous TLR9 into the LAMP1^+^ compartment.

### NaR9 inhibits TLR9 responses of BM-derived cells

**Figure 6:**
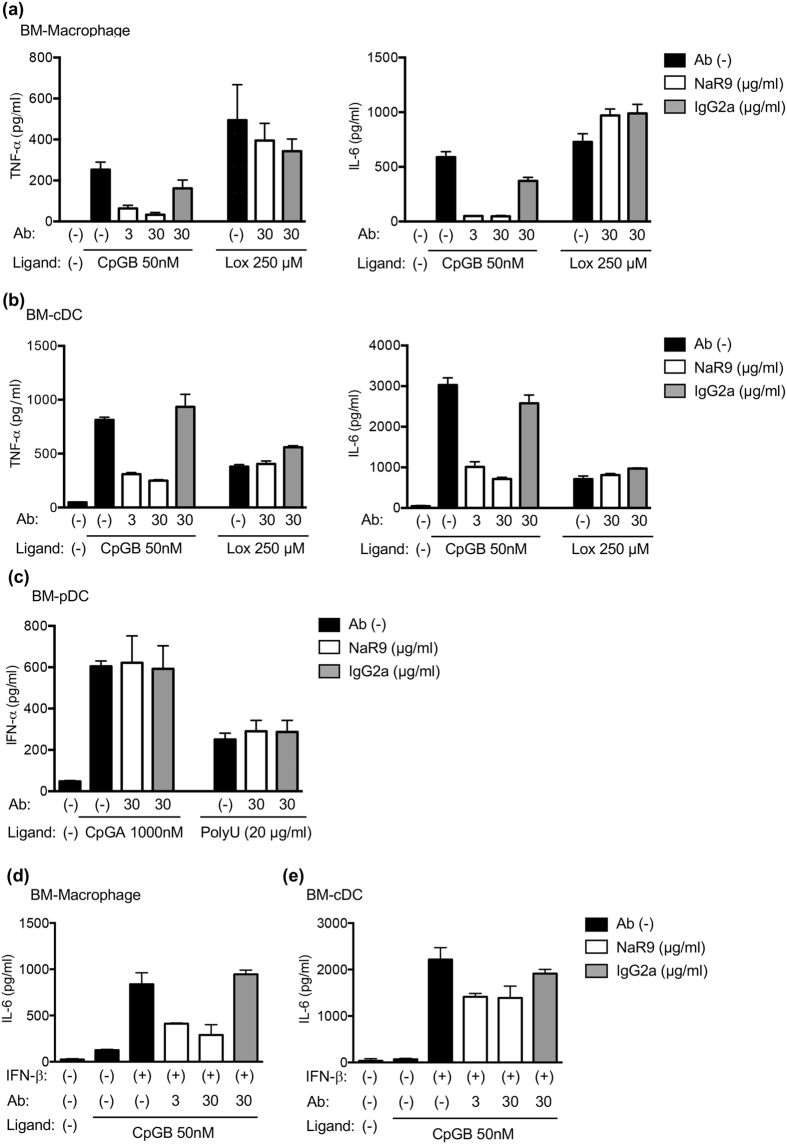
Inhibitory effect of TLR9 mAb on TLR9 responses. (**a–c**) BM-Macs (**a**), BM-cDCs (**b**) and BM-pDCs (**c**) were stimulated with CpGB or CpGA. As a control, cells were stimulated by loxoribine or poly U with DOTAP. Four hours before stimulation, the cells were treated with NaR9 or isotype-matched control mAb (IgG2a) at the indicated concentrations. Supernatant was collected after 24 h of stimulation, and cytokine production was evaluated by ELISA. (**d**,**e**) BM-Macs (**d**) and BM-cDCs (**e**) were pre-treated with medium or IFN-β (1000 U/ml) for 24 h. The cells were treated with NaR9 or isotype-matched control mAb (IgG2a) at the indicated concentrations for 4 h and stimulated by 50 nM of CpGB. Twenty-four hours after stimulation with CpGB, culture supernatants were collected, and the concentration of IL-6 was determined by ELISA. The results are represented by the mean value and the s.d. from triplicated wells. These experiments were repeated at least 3 times (**a**–**c**) or twice (**d**,**e**), and representative data are shown.

We previously reported that the anti-mouse TLR7 mAb inhibited the TLR7 response and ameliorated inflammatory disorders in *Unc93b1*^D34A/D34A^ mice^22^. Here, the effect of NaR9 on the TLR9 response was studied in bone marrow-derived Macs (BM-Macs), conventional DCs (BM-cDCs) and BM-pDCs. NaR9 drastically inhibited the production of TNF-α and IL-6 induced by CpGB but did not inhibit the response to loxoribine, a TLR7 ligand (Fig. 6a,b). In contrast to BM-Macs and BM-cDCs, TLR9-dependent IFN-α production by BM-pDCs was not impaired by NaR9 (Fig. 6c).

Next, we tested the inhibitory effect of NaR9 under severe conditions. As shown above, IFN-β enhanced the expression of TLR9 and the response to the TLR9 ligand (Fig. 4). Even with pre-treatment of IFN-β, NaR9 inhibited the production of IL-6 by BM-Macs and BM-cDCs, induced by a drastically enhanced response to CpGB (Fig. 6d,e).

**Figure 7:**
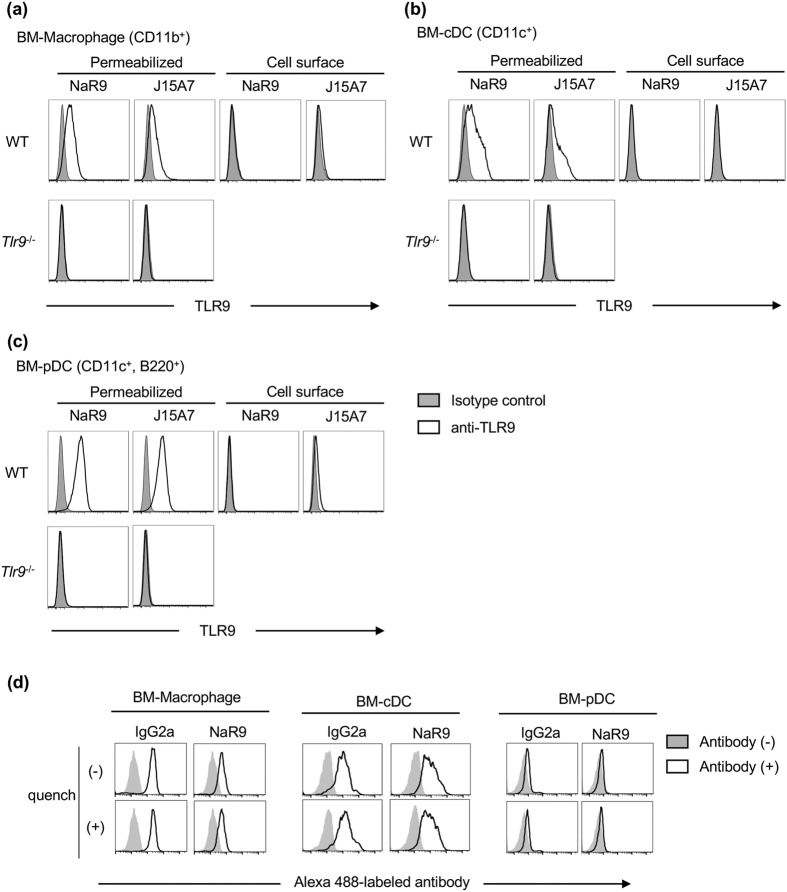
Uptake of NaR9 is required for the inhibitory effect on TLR9. (**a–c**) BM-Macs (**a**), BM-cDCs(**b**) and BM-pDCs (**c**) from WT or *Tlr9*−/− mice were stained with isotype controls (grey histograms) or anti-TLR9 mAbs (open histograms). Cells were stained with or without a membrane-permeabilizing reagent.(**d**) BM-Macs, BM-cDCs and BM-pDCs were incubated with 10 μg/ml of Alexa 488-conjugated NaR9 or isotype control IgG2a for 24 h. Engulfed antibodies were detected by flow cytometry with or without quenching of antibodies on the cell surface. (**a–d**) BM-Macs were stained by anti-CD11b, and the histograms of CD11b^+^ cells are shown. BM-cDCs were stained by anti-CD11c, and the histograms of CD11c^+^ cells are shown. BM-pDCs were stained by anti-CD11c and anti-B220, and the histograms of CD11c^+^ B220^+^ cells are shown. At least 3 mice (**a**–**c**) or 2 mice (**d**) were used for each experiment, and independent experiments were repeated at least twice. Representative data are shown.

Next, we investigated why NaR9 failed to inhibit the TLR9 response in BM-pDCs. We stained cell surface TLR9 with NaR9 and J15A7, based on the hypothesis that TLR9 on the cell surface is required for inhibition and BM-pDCs do not express TLR9 on cell surfaces. In contrast to DCs and macrophages in the spleen (Figs 2 and 3), cell surface TLR9 was hardly detected on BM-Macs, BM-cDCs and BM-pDCs (Fig. 7a–c).

Another hypothesis for the different sensitivity levels of the different cells is that sensitivity is dependent on their antibody uptake activity. BM-Macs, BM-cDCs and BM-pDCs were incubated with Alexa 488-labelled antibodies, and antibody internalization was examined by flow cytometry analysis with or without quenching of the antibody on the cell surface. The antibody was internalized by BM-Macs and BM-cDCs but not by BM-pDCs (Fig. 7d).

### NaR9 protects mice from lethal inflammation induced by CpGB and D-gal

**Figure 8:**
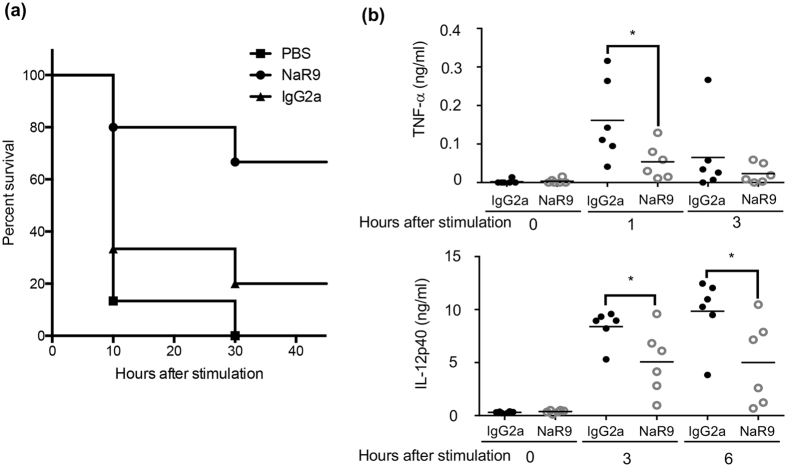
NaR9 saves mice from lethal inflammation induced by CpGB and D-gal. (**a**) C57BL/6 mice were intraperitoneally administered with anti-TLR9 or isotype-matched IgG2a mAb (25 mg/kg) 15 h before injection of CpGB (500 nmol/kg) and D-(+)-galactosamine (1 g/kg). The percentage of mice that survived the treatments is shown. Each group has 15 mice. (**b**) Blood was collected from mice at indicated time points after injection of CpGB and D-(+)-galactosamine. Serum TNF-α and IL-12p40 levels were measured by ELISA. Bars in graphs indicate the mean values. Each group has 6 mice. Statistical analysis was performed using a Student’s t-test: *p < 0.05.

Because NaR9 inhibited TLR9-dependent cytokine production in BM-Macs and BM-cDCs, the inhibitory effect of NaR9 *in vivo* was studied next. Administration of CpGB and D-(+)-galactosamine (D-gal) induces lethal fulminant hepatitis in a TLR9-dependent manner^1^,^24^, and TNF-α production in the liver is thought to cause hepatocyte cell death^24^. Mice were treated with NaR9 or isotype-matched IgG2a for 15 h and administered with CpGB and D-gal. More than 80% of mice pre-treated with PBS or isotype-matched IgG2a died within 24 h, whereas 80% of mice treated with NaR9 survived the treatment with CpGB and D-gal (Fig. 8a). To examine the effect of NaR9 on TLR9-dependent cytokine production *in vivo*, the serum concentration of TNF-α and IL-12p40 in mice treated with CpGB and D-gal was determined by ELISA. Serum TNF-α and IL-12p40 reached a peak at 1 or 3 h after CpGB and D-gal injection, respectively (Fig. 8b). NaR9 treatment significantly impaired the production of these cytokines *in vivo*. These results indicated that NaR9 rescued mice from fulminant hepatitis by inhibiting the TLR9 response *in vivo*.

## Discussion

The present study focused on endogenous TLR9 by establishing a novel mAb to mouse TLR9. Although the distribution of over-expressed, epitope-tagged TLR9 has been extensively studied^10^,^12^, it is still important to study endogenous TLR9. The present study showed that TLR9 is expressed in CD11b^+^ Ly-6G^−^ macrophages, CD11c^hi^ Siglec-H^−^ cDCs, CD11c^+^ Siglec-H^+^ pDCs and CD19^+^ B cells in the spleen. Although human granulocytes are reported to express cell surface TLR9^25^, CD11b^+^ Ly-6G^+^ mouse granulocytes in the spleen did not express TLR9. Interestingly, TLR9 was also detected on the surface of cDCs and pDCs but to a much lesser extent on B cells and monocytes. The cell surface expression of TLR9 seems to vary not only with cell types but also with the status of cell differentiation and activation. In contrast to splenic DCs, TLR9 expression was very low on the surface of BM-derived cells. Furthermore, IFN-β, but not TNF-α, enhanced TLR9 expression in a macrophage cell line RAW264.7, BM-Macs and BM-cDCs. Given the key role of type I IFN in virus infection, type I IFN-inducing TLR9 ligands is likely to contribute to the rapid induction of innate immune responses by enhancing TLR9 expression.

CpGB-induced TLR9 trafficking into the LAMP1^+^ compartment and TLR9 was detected not only in endolysosomes but also on the cell surface. It is possible that TLR9 shuttles between the cell surface and endolysosomal compartments^26^, and our results suggest that cell surface TLR9 comes from the LAMP1^+^ compartment.

Newly established anti-TLR9 mAb NaR9 inhibited TLR9-dependent cytokine production in BM-Macs and BM-cDCs but not in pDCs. The difference in the inhibitory effect of NaR9 among cells is related to the internalization of antibodies, but the mechanism by which NaR9 inhibits the TLR9 response following the internalization is unclear. One possibility is the inhibition of TLR9 binding to CpG-ODN; however, NaR9 bound to TLR9 mutants at the CpGB binding site (W47A, R74A and F108A) as much as to WT TLR9. These results did not support the possibility that NaR9 inhibits TLR9 binding to CpG-ODN. Another possibility is the inhibition of TLR9 dimerization. The extracellular domain of TLR9 consists of 26 tandem repeats of the leucine-rich repeat (LRR) protein motif 23. Proteolytic cleavage of the loop between LRR14 and LRR15 generates N-terminal and C-terminal fragments of TLR9, and the cleavage is required for ligand-dependent TLR9 dimerization. If NaR9 reacts with the loop or LRR14, it is possible that NaR9 inhibits TLR9 dimerization. These possibilities should be addressed by performing an assay to detect TLR9 dimerization.

NaR9 not only inhibited TLR9 responses in an *in vitro* assay but also rescued mice from lethal liver failure induced by the TLR9 ligand CpGB and D-gal. Because serum TNF-α and IL-12p40 induced by CpGB and D-gal was significantly decreased by the treatment with NaR9, the cells producing proinflammatory cytokines might be sensitive to NaR9. In addition to the acute inflammation studied here, it is reported that chronic inflammation in liver such as non-alcoholic steatohepatitis (NASH) is driven by TLR9^27^. Mitochondrial DNA (mtDNA) from hepatocytes activates immune cells, leading to chronic hepatitis and cirrhosis. Given that anti-TLR7 mAb rescues *Unc93b1*^D34A/D34A^ mice from chronic hepatitis^22^, anti-TLR9 mAb is also expected to cure TLR9-dependent chronic diseases.

Compared with the other reagents inhibiting the response of TLR9, the use of an antibody as a therapeutic intervention has advantages and disadvantages. First, the inhibitory effect of anti-TLR9 is dependent on the uptake activity of the cells. This might be a disadvantage if the corresponding cells do not have antibody uptake activity, but has the advantage of specificity if the corresponding cells do have uptake activity. Next, antibodies have much higher specificity than small chemicals or short ODNs. Some of these reagents not only inhibit the response of TLR9 but also inhibit the response of TLR7^28^,^29^,^30^. It is advantageous to use these reagents if the targeting diseases are driven by both TLR7 and TLR9; however, specificity is important for medicine to avoid unexpected results when being used as a therapeutic intervention. In conclusion, the present study suggests that the anti-TLR9 mAb is a promising tool for therapeutic intervention in TLR9-dependent inflammatory diseases.

## Methods

### Generation of anti-mouse TLR9 monoclonal antibody

To establish an anti-mouse TLR9 monoclonal antibody (mAb), *Tlr9*^*−*/*−*^ mice on a BALB/c background were immunized by intraperitoneally administering Ba/F3 cells expressing mouse TLR9 (Ba/F3-mTLR9) with complete Freund’s adjuvant/incomplete Freund’s adjuvant as adjuvants. Five days after the final immunization, splenic cells were fused with SP2/O myeloma cells. A hybridoma producing anti-TLR9 mAb was selected by flow cytometry staining of Ba/F3-mTLR9. A subclass of this mAb was determined as IgG2a/κ and named NaR9.

### Mice

C57BL/6 mice were purchased from Japan SLC, Inc. (Shizuoka, Japan). *Tlr9*^*−*/*−*^ mice on a C57BL/6 background were generated in our laboratory. *Tlr9*^*−*/*−*^ mice on a BALB/c background were backcrossed seven times with BALB/c wild-type mice purchased from Japan SLC, Inc. All animal experiments were approved by the Animal Research Committee of the Institute of Medical Science, The University of Tokyo, and performed in accordance with the guidelines.

### Reagents and antibodies

Anti-TLR9 mAbs, NaR9, J15A7, B33A4 and C34A1, were purified from ascitic fluid as shown previously^12^. Streptavidin-PE, anti-mouse IgG1-PE, anti-mouse IgG2a-PE, isotype control antibodies (mouse IgG1, mouse IgG2a), anti-mouse CD16/32, anti-mouse CD19-APC-Cy7, anti-mouse CD11b-APC, anti-mouse CD11c-APC, anti-mouse CD11c-PE-Cy7, anti-mouse Siglec-H-FITC and anti-mouse Ly-6G-PerCP-Cy5.5 were purchased from BioLegend (San Diego, CA, USA). Anti-mouse B220-APC was purchased from TONBO biosciences (San Diego, CA, USA). J15A7-PE, anti-mouse CD49b-BV421 and anti-mouse CD11b-BV510 were purchased from BD Biosciences (Franklin Lakes, NJ, USA). Anti-Calnexin and anti-LAMP1 were purchased from Abcam. Anti-LAMP2 was purchased from eBiosciences (San Diego, CA, USA). CpGA 1585 (5′-G*G*GGTCAACGTTGAG*G*G*G*G*G-3′, asterisks indicate phosphorothioated residues), PolyU (5′-UUUUUUUUU UUUUUUUUUU-3′, whole phosphorothioated) and CpGB 1688 (5′-TCCATGACGTTCCTGATGCT-3′, whole phosphorothioated) were synthesized by FASMAC (Atsugi, Japan). Loxoribine (7-allyl-7,8-dihydro8-oxo-guanosine) was purchased from Enzo Life Science (Farmingdale, NY, USA). Saponin and D-(+)-galactosamine were purchased from Sigma-Aldrich (St. Louis, MO, USA). FuGene6 and DOTAP were purchased from Roche Applied Science (Basel, Switzerland).

### Cell culture

RAW264.7 cells were cultured in high-glucose DMEM supplemented with 10% foetal bovine serum (FBS), penicillin–streptomycin–glutamine (PS/Gln, GIBCO, Waltham, MA, USA) and 50 μM 2-ME. Ba/F3 cells were cultured in Roswell Park Memorial Institute (RPMI) 1640 medium supplemented with IL-3, 10% FBS, PS/Gln and 50 μM 2-ME. Bone marrow-derived macrophages (BM-Macs), conventional DCs (cDCs) or plasmacytoid DCs (pDCs) were prepared. In brief, to induce macrophages, BM cells were plated at 1 × 10^7^ cells per 10 ml with 10% FCS–RPMI1640 supplemented with 100 ng/ml of recombinant M-CSF (PeproTech, Rocky Hill, NJ, USA) in 10-cm cell culture dishes for 6 days. To induce cDCs or pDCs, BM cells were plated at 1 × 10^7^ cells per 10 ml with 10% FCS–RPMI1640 supplemented with 10 ng/ml of recombinant GM-CSF or Flt3-L (PeproTech) in 10-cm cell culture dishes for 7 days.

### Plasmid constructs and retrovirus transduction

TLR9 was amplified by PCR and cloned into retroviral pMX vectors (kindly provided by Dr Kitamura, The University of Tokyo). For the constructs encoding TLR9, N-fragments (1–454 amino acids, a.a.) were cloned into the pMX vector. For the TLR9C constructs, TLR9 fragments corresponding to amino acids 1–35, the signal peptide and following 10 amino acids, fused to amino acids 455–818 (TLR9C deletion 36–454), were cloned into the pMX vector. The In-Fusion HD cloning kit (TaKaRa Bio, Kusatsu, Japan) and Rapid DNA Ligation kit (Roche Applied Science) were used for cloning. TLR9 mutants, W47A, R74A, F108A, H642A, F668A and N695A, were cloned into the pMX-IRES rat CD2 vector.

Plasmids were transfected into Plat-E packaging cells 1 × 10^5^ per well with polyethylenimine (Polysciences, Inc., Warrington, PA, USA) or FuGene6. After 2 days of incubation, supernatants were collected as virus suspensions. Ba/F3 cells were transduced by virus suspensions mixed with DOTAP.

### Cell staining and flow cytometry

Ba/F3 cells or RAW264.7 cells were collected and stained with anti-TLR9 mAbs. The Fc receptor on RAW264.7 cells was blocked by anti-CD16/32. To detect TLR9 on the cell surface, a staining buffer (1x PBS, 2% FBS, 2 mM EDTA and 0.1% sodium azide) was used. For membrane permeabilization, 0.1% saponin was added to the staining buffer. Cells were incubated in 100 ng/ml of purified anti-TLR9, 500 ng/ml of biotin-conjugated or 50 ng/ml of PE-conjugated antibody for 20 min at 4 °C. After 2 wash cycles, cells were incubated in 200 ng/ml of PE-conjugated anti-IgG1 (for J15A7), anti-IgG2a (for NaR9, B33A4 and C34A1) or 50 ng/ml of streptavidin. The cells were washed twice and suspended in staining buffer for flow cytometry.

For staining of splenocytes, a single-cell suspension was prepared from the spleen, and red blood cells were lysed in RBC lysis buffer (BioLegend). The Fc receptor on splenocytes was blocked by anti-CD16/32, and the cells were subjected to counter staining. CD19, CD11c and Siglec-H were stained for separation of B cells, cDCs and pDCs. CD49b, CD11b, CD11c and Ly6G were stained for separation of monocytes. After counter staining, the cells were fixed by a Fixation/Permeabilization buffer (BD) for 20 min at 4 °C. The cells were washed with 1x Perm/Wash buffer (BD) and incubated in 2000 ng/ml of anti-TLR9 in 1x Perm/Wash buffer for 30 min at 4 °C. The cells were washed twice with 1x Perm/Wash buffer and incubated in 200 ng/ml of anti-IgG1 or anti-IgG2a for 30 min at 4 °C. Finally, the cells were washed twice with 1x Perm/Wash buffer and suspended in a staining buffer for flow cytometry. To stain TLR9 on the cell surface, a staining buffer was used instead of 1x Perm/Wash buffer under non-fixing conditions.

This protocol was also used to stain BM-derived cells. For counter staining, BM-Macs were stained with anti-CD11b. BM-cDCs were stained with anti-CD11c. BM-pDCs were stained with anti-CD11c and anti-B220.

Prepared cells were subjected to flow cytometry analysis by LSRFortessa X-20 (BD). For detection of PE, a yellow-green laser was used. Flow cytometry data were analysed using FlowJo software (FlowJo, Ashland, OR, USA).

### Confocal microscopy

RAW264.7 cells were allowed to adhere and were stimulated with or without 500 nM of CpGB. Twenty-four hours later, the cells were then fixed in 4% paraformaldehyde for 3 min and permeabilized with 0.1% saponin in 25% Blocking One (Nacalai, Kyoto, Japan) for 20 min. The cells were incubated with anti-TLR9 (1 μg/ml NaR9) for 20 min at RT. To stain organelle markers, anti-Calnexin, anti-LAMP1 or anti-LAMP2 was incubated with anti-TLR9. The incubated cells were washed and then incubated with Alexa Fluor 488- or 568-conjugated secondary antibodies (Thermo Fisher Scientific, Waltham, MA, USA) for 20 min at RT. The immunostained RAW264.7 cells were analysed using LSM710 confocal microscope with a 63x NA1.4 Plan-Apochromat oil immersion lens (Carl Zeiss Microscopy). Fluorescent images were acquired and analysed using LSM710 ZEN software (Carl Zeiss Microscopy, Oberkochen, Germany). The co-localization of TLR9 with Calnexin, LAMP1 or LAMP2 was analysed using LSM710 ZEN software according to the manufacturer’s manual. Pearson’s correlation coefficients were used for the statistical analysis of co-localization. A Pearson’s coefficient of <0.2 represents poor co-localization.

### Ligand stimulation and ELISA

RAW264.7 cells, BM-Macs or cDCs (2 × 10^5^ cells/well) were seeded in 24-well plates and stimulated with Lipid A, CpGB, IFN-β or TNF-α for 24 h. The cells were subjected to TLR9 staining and analysed using LSRFortessa X-20.

RAW264.7 cells (2 × 10^4^ cells/well) were seeded in 96-well plates and treated with medium, IFN-β or TNF-α. Twenty-four hours later, the cells were stimulated with CpGB or CpGA for 24 h. RANTES in the supernatant was measured using a DuoSet ELISA Development System (R&D Systems, Minneapolis, MN, USA). BM-Macs, cDCs or pDCs (5 × 10^4^ cells/well) were plated in 96-well plates, and treated with medium, NaR9 or IgG2a control Ab. Four hours later, the treated cells were stimulated by CpGB, CpGA, loxoribine or PolyU (with DOTAP) for 24 h. TNF-α and IL-6 in the supernatant were measured using Ready-SET-Go! ELISA Sets (eBiosciences), and IFN-α was measured using a VeriKine ELISA Kit (PBL Assay Science, Piscataway, NJ, USA).

### Antibody-uptake assay

BM-Macs, BM-cDCs or BM-pDCs (1 × 10^6^ cells/well) were seeded in 6-well plates and incubated with 10 μg/ml of NaR9 or IgG2a control Ab conjugated to Alexa 488 (Alexa Fluor^®^ 488 Antibody Labeling Kit, Thermo Fisher) at 37 °C for 24 h. The cells were collected and treated with/without anti-Alexa488 pAb (Thermo Fisher) for quenching cell surface fluorescence. The prepared cells were subjected to flow cytometry analysis using LSRFortessa X-20.

### *In vivo* administration of CpGB and D-(+)-galactosamine

C57BL/6 WT mice were treated intraperitoneally with the indicated mAbs or PBS. Fifteen hours later, mice were administered intraperitoneally with 10 nanomoles of CpGB and 20 mg of D-(+)-galactosamine per head. Blood samples were collected from mice at 0, 1, 3 and 6 h after stimulation, and separated serum was subjected to ELISA to measure the production of TNF-α and IL12-p40.

### Statistical analysis

Statistical significance was calculated by performing a two-tailed unpaired Student’s t-test. A *p* value of <0.05 was considered statistically significant.

## Additional Information

**How to cite this article**: Murakami, Y. *et al*. The protective effect of the anti-Toll-like receptor 9 antibody against acute cytokine storm caused by immunostimulatory DNA. *Sci. Rep.* **7**, 44042; doi: 10.1038/srep44042 (2017).

**Publisher's note:** Springer Nature remains neutral with regard to jurisdictional claims in published maps and institutional affiliations.

## References

[b1] Hemmi H. . A Toll-like receptor recognizes bacterial DNA. Nature 408, 740–745, doi: 10.1038/35047123 (2000). 11130078

[b2] Marshak-Rothstein A. & Rifkin I. R. Immunologically active autoantigens: the role of toll-like receptors in the development of chronic inflammatory disease. Annu Rev Immunol 25, 419–441 (2007). 17378763 10.1146/annurev.immunol.22.012703.104514

[b3] Lande R. . Plasmacytoid dendritic cells sense self-DNA coupled with antimicrobial peptide. Nature 449, 564–569 (2007). 17873860 10.1038/nature06116

[b4] Leadbetter E. A. . Chromatin-IgG complexes activate B cells by dual engagement of IgM and Toll-like receptors. Nature 416, 603–607 (2002). 11948342 10.1038/416603a

[b5] Marshak-Rothstein A. . Comparison of CpG s-ODNs, chromatin immune complexes, and dsDNA fragment immune complexes in the TLR9-dependent activation of rheumatoid factor B cells. J Endotoxin Res 10, 247–251, doi: 10.1179/096805104225005850 (2004). 15373969

[b6] Viglianti G. A. . Activation of autoreactive B cells by CpG dsDNA. Immunity 19, 837–847 (2003). 14670301 10.1016/s1074-7613(03)00323-6

[b7] Christensen S. R. . Toll-like receptor 7 and TLR9 dictate autoantibody specificity and have opposing inflammatory and regulatory roles in a murine model of lupus. Immunity 25, 417–428, doi: 10.1016/j.immuni.2006.07.013 (2006). 16973389

[b8] Zhu F. G. . A novel antagonist of Toll-like receptors 7, 8 and 9 suppresses lupus disease-associated parameters in NZBW/F1 mice. Autoimmunity 46, 419–428, doi: 10.3109/08916934.2013.798651 (2013). 24083389

[b9] Jiang W. . A Toll-Like Receptor 7, 8, and 9 Antagonist Inhibits Th1 and Th17 Responses and Inflammasome Activation in a Model of IL-23-Induced Psoriasis. J Invest Dermatol 133, 1777–1784, doi: 10.1038/jid.2013.57 (2013). 23370539

[b10] Sasai M., Linehan M. M. & Iwasaki A. Bifurcation of Toll-Like Receptor 9 Signaling by Adaptor Protein 3. Science 329, 1530–1534, doi: 10.1126/science.1187029 (2010). 20847273 PMC3063333

[b11] Guiducci C. . Properties regulating the nature of the plasmacytoid dendritic cell response to Toll-like receptor 9 activation. J Exp Med 203, 1999–2008 (2006). 16864658 10.1084/jem.20060401 PMC2118381

[b12] Latz E. . TLR9 signals after translocating from the ER to CpG DNA in the lysosome. Nat Immunol 5, 190–198 (2004). 14716310 10.1038/ni1028

[b13] Honda K. . Spatiotemporal regulation of MyD88-IRF-7 signalling for robust type-I interferon induction. Nature 434, 1035–1040 (2005). 15815647 10.1038/nature03547

[b14] Onji M. . An essential role for the N-terminal fragment of Toll-like receptor 9 in DNA sensing. Nat Commun 4, 1949, doi: 10.1038/ncomms2949 (2013). 23752491

[b15] Kanno A. . Essential role for Toll-like receptor 7 (TLR7)-unique cysteines in an intramolecular disulfide bond, proteolytic cleavage and RNA sensing. Int Immunol 25, 413–422, doi: 10.1093/intimm/dxt007 (2013). 23446849

[b16] Murakami Y. . Roles of the cleaved N-terminal TLR3 fragment and cell surface TLR3 in double-stranded RNA sensing. J Immunol 193, 5208–5217, doi: 10.4049/jimmunol.1400386 (2014). 25305318

[b17] Fukui R. . Unc93B1 biases Toll-like receptor responses to nucleic acid in dendritic cells toward DNA- but against RNA-sensing. J Exp Med 206, 1339–1350 (2009). 19451267 10.1084/jem.20082316 PMC2715051

[b18] Brinkmann M. . The interaction between the ER membrane protein UNC93B and TLR3, 7, and 9 is crucial for TLR signaling. J Cell Biol 177, 265–275 (2007). 17452530 10.1083/jcb.200612056 PMC2064135

[b19] Kim Y., Brinkmann M., Paquet M. & Ploegh H. UNC93B1 delivers nucleotide-sensing toll-like receptors to endolysosomes. Nature 452, 234–238 (2008). 18305481 10.1038/nature06726

[b20] Tabeta K. . The Unc93b1 mutation 3d disrupts exogenous antigen presentation and signaling via Toll-like receptors 3, 7 and 9. Nat Immunol 7, 156–164 (2006). 16415873 10.1038/ni1297

[b21] Fukui R. . Unc93B1 restricts systemic lethal inflammation by orchestrating Toll-like receptor 7 and 9 trafficking. Immunity 35, 69–81, doi: 10.1016/j.immuni.2011.05.010 (2011). 21683627

[b22] Kanno A. . Targeting cell surface TLR7 for therapeutic intervention in autoimmune diseases. Nat Commun 6, 6119, doi: 10.1038/ncomms7119 (2015). 25648980

[b23] Ohto U. . Structural basis of CpG and inhibitory DNA recognition by Toll-like receptor 9. Nature 520, 702–705, doi: 10.1038/nature14138 (2015). 25686612

[b24] Sparwasser T. . Macrophages sense pathogens via DNA motifs: induction of tumor necrosis factor-alpha-mediated shock. Eur J Immunol 27, 1671–1679, doi: 10.1002/eji.1830270712 (1997). 9247576

[b25] Lindau D. . Primary blood neutrophils express a functional cell surface Toll-like receptor 9. Eur J Immunol 43, 2101–2113, doi: 10.1002/eji.201142143 (2013). 23686399

[b26] Lee B. L. . UNC93B1 mediates differential trafficking of endosomal TLRs. Elife 2, e00291, doi: 10.7554/eLife.00291 (2013). 23426999 PMC3576711

[b27] Garcia-Martinez I. . Hepatocyte mitochondrial DNA drives nonalcoholic steatohepatitis by activation of TLR9. J Clin Invest 126, 859–864, doi: 10.1172/JCI83885 (2016). 26808498 PMC4767345

[b28] Lamphier M. . Novel small molecule inhibitors of TLR7 and TLR9: mechanism of action and efficacy *in vivo*. Mol Pharmacol 85, 429–440, doi: 10.1124/mol.113.089821 (2014). 24342772

[b29] Pawar R. D. . Inhibition of Toll-like receptor-7 (TLR-7) or TLR-7 plus TLR-9 attenuates glomerulonephritis and lung injury in experimental lupus. J Am Soc Nephrol 18, 1721–1731, doi: ASN.2006101162 [pii]10.1681/ASN.2006101162 (2007). 17460144 10.1681/ASN.2006101162

[b30] Barrat F. J., Meeker T., Chan J. H., Guiducci C. & Coffman R. L. Treatment of lupus-prone mice with a dual inhibitor of TLR7 and TLR9 leads to reduction of autoantibody production and amelioration of disease symptoms. Eur J Immunol 37, 3582–3586, doi: 10.1002/eji.200737815 (2007). 18034431

